# Supplementary material for: School Nurse Perspectives of Working with Children and Young People in the United Kingdom during the COVID-19 Pandemic: An Online Survey Study
Source: Int J Environ Res Public Health. 2022 Dec 28;20(1):481. doi: 10.3390/ijerph20010481 (PMC9819616; doi:10.3390/ijerph20010481)
Supplement: Supplementary file 1 [file ijerph-20-00481-s001.zip › ijerph-2012199-supplementary.pdf]

File S1: The final survey.

Learning from school nursing practices and new ways of working during the COVID-19 pandemic: Online survey

Mandatory Inclusion / Exclusion Question - Do you work as a school nurse [school health nurse]?
Yes [continue to questionnaire Q1]
No [We thank you for your time spent taking this survey. Your response has been recorded]

Section 1 - Background information

1. What type of school(s) do you work with (please tick all that apply)

State
Independent
Special needs
Other

2. What age are the children you work with? (please tick all that apply)

Primary
Secondary
Further education college (under 18s)

3. Which of the statements below best describes the way you work with schools? (please tick all that apply)

I am attached to one school
I am attached to one school and am the main school nurse
I am attached to named schools
I am attached to named schools and am the main school nurse
I am part of a school nursing team who share responsibility for the schools in our area

4. Who provides the School Nursing service?

Local Authority
Private
Charity
School
Other, please specify [open text box]

5. Please could you provide the first part (the letters and numbers before the space) of the postcode of your place of work, e.g., if your postcode is OX3 0FL, type OX3. If you work in more than one location, please enter your hub postcode:

[open text box]

6. What are your contracted hours as a school nurse?

Full time – all year
Full time – term time only
Part time – all year
Part time – term time only
Other (please specify):

7. What is your nursing field of registration (please tick all that apply)

Adult
Child
Mental health
Learning disability
Midwifery

8. How many years have you been working as a school nurse?

[open text box]

9. Do you have the Specialist Community Public Health Nursing (SCPHN) school nurse qualification)?

Yes (SN)
Yes (HV)
Yes (SN and HV)
No

9.2 If yes. What year did you qualify as a SCPHN school nurse?

[open text box]

Section 2 - Working during the pandemic

10. Were you redeployed during the COVID-19 pandemic?

Yes
No

10.2 If yes. How many weeks was this for?

[open text box]

10.3 If yes. Where were you redeployed to?

[open text box]

11. Did your workload change during the COVID-19 pandemic?

No
Yes, it decreased
Yes, it increased

12. Did you experience a change in children's, young people's or families' contact with the school nursing service during COVID-19:

No change in contact
Decreased contact
Increased contact

12.2 If change (increase/decrease) reported. Can you briefly describe any change(s)?

[open text box]

13. As a result of the COVID-19 pandemic and the restrictions to in-person contact, how did the methods you used to communicate with children, young people and families, using the following modes of service delivery, change?

Response options: Increased/same/Decreased/never used/Not applicable
Telephone consultations
Email
Online/virtual consultations
Online classroom sessions
Virtual nurses office
Chat health
Consultations outside, e.g., 'walk and talk'
Short health promotion videos
Apps (such as ChatHealth)
Other, please specify [open text box]

14. As a result of the COVID-19 pandemic and the restrictions to in-person contact, how did the way you communicated with the multi-disciplinary team, using the following methods of communication, change?

Response options: Increased/same/less/Never used/Not applicable
Telephone
Email
Texting/WhatsApp
Online/virtual meetings
Other, please specify [open text box]

15. Have you had any feedback on, or conducted any evaluation of the school nursing services offered during COVID-19?

Yes
No

15.2 Please can you provide a summary of the feedback received or evaluation conducted on the school nursing services offered:

[open text box]

16. Did you experience any particular challenges or barriers to perform your school nurse role?

[open text box]

Section 3 - Working with vulnerable children

17. Did COVID-19 restrictions impact your ability to identify vulnerable children, young people and families?

Yes
No

18. Did COVID-19 restrictions impact your ability to provide support to vulnerable children, young people and families that were already known to you?

Yes
No

18.2 If yes. Can you give a brief description of how COVID-19 restrictions impacted your ability to work with vulnerable children, young people and families that were already known to you?

[open text box]

19. Overall, considering the impact of lockdown and the resulting changes in workload, what has been the impact of COVID-19 on school nursing partnership working (e.g., with education, social care, community health services, emergency departments, sexual health services, child and adolescent mental health services, community children's nursing teams, police services, substance misuse services, etc.)?

It improved
It stayed the same
It was harder
It was variable

19.2 Can you give some examples of where school nursing partnership working has improved?

[open text box]

19.3 Can you give some examples of where school nursing partnership working has been harder or more challenging?

[open text box]

20. Do you have anything else to add about your experience of being a school nurse during the pandemic and its impact on your practice?

[open text box]

Next phase of this research study

We would like to speak to school nurses about their experience of practising during the COVID-19 pandemic. We would like to explore in more detail how practice was impacted specifically with vulnerable children and young people. What were the challenges? Were new ways of working needed? Have these been effective? Will you carry on with these new ways of working beyond the pandemic?

We will be organising a range of ways to hear your accounts that can fit your busy schedule such as focus groups, one-to-one video or telephone interviews or focus groups.

Would you be interested in taking part?

Yes
No

If yes. Please add your email here [independent google form] and we will be in touch with more information about the next phase of the study in due course.

Thank you for taking the time to complete this survey. Please submit your responses by clicking the arrow in the bottom right corner.

If this survey raises any professional or safeguarding issues for you, please contact your supervisor/line manager or the designated safeguarding lead in your organisation. Support can also be sought from: NSPCC safeguarding helpline (0808 800 5000).
